# Noncoding RNA in the transcriptional landscape of human neural progenitor cell differentiation

**DOI:** 10.3389/fnins.2015.00392

**Published:** 2015-10-23

**Authors:** Patrick M. Hecht, Inmaculada Ballesteros-Yanez, Nicole Grepo, James A. Knowles, Daniel B. Campbell

**Affiliations:** ^1^Keck School of Medicine, Zilkha Neurogenetic Institute, University of Southern CaliforniaLos Angeles, CA, USA; ^2^Department of Inorganic, Organic Chemistry and Biochemistry, Faculty of Medicine, CRIB, University of Castile-La ManchaCiudad Real, Spain; ^3^Department of Psychiatry and the Behavioral Sciences, Keck School of Medicine, University of Southern CaliforniaLos Angeles, CA, USA

**Keywords:** noncoding RNA, neuronal progenitor, neural differentiation, RNA-sequencing, WGCNA

## Abstract

Increasing evidence suggests that noncoding RNAs play key roles in cellular processes, particularly in the brain. The present study used RNA sequencing to identify the transcriptional landscape of two human neural progenitor cell lines, SK-N-SH and ReNcell CX, as they differentiate into human cortical projection neurons. Protein coding genes were found to account for 54.8 and 57.0% of expressed genes, respectively, and alignment of RNA sequencing reads revealed that only 25.5–28.1% mapped to exonic regions of the genome. Differential expression analysis in the two cell lines identified altered gene expression in both protein coding and noncoding RNAs as they undergo neural differentiation with 222 differentially expressed genes observed in SK-N-SH cells and 19 differentially expressed genes in ReNcell CX. Interestingly, genes showing differential expression in SK-N-SH cells are enriched in genes implicated in autism spectrum disorder, but not in gene sets related to cancer or Alzheimer's disease. Weighted gene co-expression network analysis (WGCNA) was used to detect modules of co-expressed protein coding and noncoding RNAs in SK-N-SH cells and found four modules to be associated with neural differentiation. These modules contain varying levels of noncoding RNAs ranging from 10.7 to 49.7% with gene ontology suggesting roles in numerous cellular processes important for differentiation. These results indicate that noncoding RNAs are highly expressed in human neural progenitor cells and likely hold key regulatory roles in gene networks underlying neural differentiation and neurodevelopmental disorders.

## Introduction

Neural differentiation is a complex biological process requiring precise regulation of gene expression. However, the molecular mechanisms underlying this transcriptional control remain largely unknown. Recent advances in genomics technology have unveiled the complexities of the mammalian transcriptome. It is now understood that most of the genome is transcribed with less than 2% encoding for protein resulting in a vast and largely uncharacterized landscape of non-protein coding RNAs (Carninci et al., 2005; Birney et al., 2007; Kapranov et al., 2007, 2010; Derrien et al., 2012; Djebali et al., 2012; Dunham et al., 2012). Once thought to be transcriptional noise, these noncoding RNAs are emerging as key regulatory elements of gene expression. While several noncoding RNAs have been shown to be important in various biological processes, including cell differentiation (Morris and Mattick, 2014), the functions of most noncoding RNA transcripts are unknown.

Previous studies investigating the molecular dynamics of neural differentiation have focused on the role of protein coding genes while largely ignoring noncoding RNAs (Shin et al., 2007; Wu et al., 2010; Fathi et al., 2011). Additionally, many of these studies have used cells generated from smaller animals such as rodents (Ahn et al., 2004; Gurok et al., 2004; Aiba et al., 2006; Lee et al., 2006; Suh et al., 2008). It has been shown that the transcriptional landscape of more evolutionarily complex organisms contain larger numbers of noncoding RNAs (Taft et al., 2007). In fact, humans have been shown to express one of the largest quantities of noncoding RNAs (Liu et al., 2013) with these transcripts showing dynamic expression patterns in the developing brain (Lipovich et al., 2014; Ziats and Rennert, 2014). Therefore, it is likely that these non-protein coding transcripts play key regulatory roles in the neurodevelopmental processes contributing to the complexities observed in the human brain. Additionally, alterations in the expression patterns of noncoding RNAs have been suggested to play critical roles in the underlying pathophysiology of neuropsychiatric disorders (Takahashi et al., 2003; Kerin et al., 2012; Ziats and Rennert, 2013; Barry et al., 2014).

Human neural progenitor cells serve as promising models for investigating the mechanisms underlying early neurodevelopmental processes such as differentiation. Two such cell lines are SK-N-SH and ReNcell CX cells. SK-N-SH cells were derived from human neuroblastoma cells collected from a 4 year-old female. These cells have the ability to differentiate into a neuronal phenotype characterized by extensive neurite outgrowth. ReNcell CX cells were derived from a human 14 week fetal cerebral cortex and immortalized by retroviral MYC oncogene transduction. ReNcell CX human neural progenitor cells express high levels of the neural stem cell markers *Nestin* and *Sox2* and differentiate into *TuJ1*-positive neurons. Therefore, these cell lines are ideal for studying the complex molecular mechanisms underlying human neural differentiation. The present study used RNA sequencing on these two neural progenitor cell lines to gain insight into the transcriptional profile of human neural differentiation. In order to identify noncoding RNAs possibly regulating this biological process, a weighted gene co-expression network was constructed to form clusters (modules) of protein coding and non-protein coding genes showing highly correlated expression patterns. Therefore, the gene networks regulated by noncoding RNAs can then be inferred through the functional properties of their co-expressed protein coding genes.

## Materials and methods

### Cell culture

The human neural progenitor cell lines, SK-N-SH and ReNcell CX, were used to measure gene expression as they differentiate into cortical projection neurons. SK-N-SH cells (American Type Culture Collection; Manassas, VA, USA) were maintained in a minimum essential medium supplemented with 10% heat-inactivated fetal bovine serum, 1% penicillin/streptomycin, nonessential amino acids, and 1.5 g l^−1^ sodium bicarbonate in 183 cm flasks at 37°C and 5% CO_2_. ReNcell CX cells (Millipore; Billerica, MA, USA) were maintained in ReNcell Neural Stem Cell Maintenance Medium supplemented with fibroblast growth factor (FGF) and epidermal growth factor (EGF) (Millipore) in laminin-coated T75 flasks at 37°C and 5% CO_2_. Both cell lines were seeded into six 10-cm well plates and grown for 24 h (~70% confluency). All cells in this study were used as empty vector controls for other experiments. As such, cells were then transfected with 2 μg of a pIRES2-AcGFP empty vector (Clontech; Mountain View, CA, USA) using Amaxa Nucleofector technology (Lonza; Basel, Switzerland) according to the manufacturer's protocol. SK-N-SH cells were then allowed to replicate and undergo differentiation until being harvested either 24 h (*n* = 8) or 72 h (*n* = 8) following transfection with the empty vector. Similarly, ReNcell CX cells were harvested 24 h (*n* = 8) and 72 h (*n* = 8) post empty vector transfection. Prior to harvesting, cells were imaged using an Olympus CKX41 inverted microscope with an attached Q Imaging QICAM Fast 1394 Digital Camera. Each harvesting of cells was treated as an individual experiment.

### Quantitative PCR

To ensure that transfection with the empty vector did not have an overall impact on transcription, quantitative PCR (qPCR) was performed on protein coding and noncoding RNAs either showing differential expression or with known functional roles in neuronal development. SK-N-SH and ReNcell CX cells were processed as described above with the exception of a subset of cells not exposed to Nucleofector transfection. Cell pellets of untransfected SK-N-SH (24 h, *n* = 4; 72 h, *n* = 4) and ReNcell CX cells (24 h, *n* = 4; 72 h, *n* = 3) were processed for RNA isolation using Qiagen RNeasy kit (Qiagen) along with cell pellets of transfected SK-N-SH (24 h, *n* = 3; 72 h, *n* = 3) and ReNcell CX cells (24 h, *n* = 4; 72 h, *n* = 3). Superscript III (Life Technologies; Grand Island, NY, USA) was used for reverse transcription of cDNA libraries. Quantitative PCR (qPCR) was performed using the Taqman Gene Expression qPCR kits (Life Technologies) on a Life Technologies OneStepPlus real-time PCR machine. Each sample was run in triplicate along with the housekeeping gene, *GAPDH*. Assays (Life Technologies) used in this study include: *ATF3* (Hs00231069_m1), *CNTNAP2* (Hs01034283_m1), *GAPDH* (Hs99999905_m1), *HMOX1* (Hs01110250_m1), *MALAT1* (Hs00273907_s1), *MIR137* (Hs04231500_s1), *MSN* (AIWR1S8), and *PLAUR* (Hs00958880_m1). Relative quantity of the transcript was determined using the 2^−ΔΔCt^ method using *GAPDH* as a reference (Schmittgen and Livak, 2008). The observed qPCR changes in the transfected cells were compared to the cells not exposed to Nucleofector transfection using the Pearson correlation test carried out on the R statistical environment using the (cor.test) package.

### RNA-sequencing

Harvested cells were homogenized with QIAshredder spin columns (Qiagen; Valencia, CA, USA). Total cellular RNA was extracted using Qiagen RNeasy kit according to manufacturer's instructions. RNA quality was assessed using Agilent Technologies 2200 TapeStation Instrument (Agilent Technologies; Santa Clara, CA, USA) and 260/280 absorbance ratios were captured with a NanoDrop ND-1000 Spectrophotometer (Thermo Fisher Scientific; Waltham, MA, USA). cDNA was made using the Illumina Truseq Stranded Total RNA Sample Preparation kit per the manufacturer's instructions and Ribozero (Illumina; San Diego, CA, USA) was used to deplete rRNA from the sample. Libraries were multiplexed using the Illumina Truseq Stranded Total RNA Sample Preparation kit with four samples per lane, pooled and sequenced using the HiSeq2000 sequencer to generate 101 bp single-end reads (Ilumina; San Diego, CA, USA). Sequencing obtained an average depth of about 28.5 million reads per replicate in SK-N-SH cells and about 36.3 million reads per replicate in ReNcell CX cells (Supplementary Table 1). RNA sequencing data files were deposited into the SRA database under the accession number SRP064264.

### RNA-sequencing analysis

Quality control processing of raw reads was carried out using Cutadapt (version 1.3; Martin, 2011). One SK-N-SH sample collected at 72 h did not meet quality control standards and was removed from further analysis. Reads were then aligned to the ENSEMBL GrCH38 version 77 transcriptome using Bowtie2 (version 2.1.0) and TopHat (version 2.0.10; Trapnell et al., 2009). Gene annotations were derived from the ENSEMBL GrCH38.77 database. The Picard command line tool “CollectRnaSeqMetrics” (https://broadinstitute.github.io/picard/) (version 1.134) was used to assess the percentage of reads mapping to exonic, intronic, untranslated regions (UTR), and intergenic regions of the genome in both cell lines. Differential gene expression analysis was carried out using Cuffdiff (version 2.2.1; Trapnell et al., 2012). All default settings were used during the Cuffdiff analysis with the exception of masking all ribosomal RNAs and using the no-effective-length-correction option. Genes were defined as being expressed if they showed a normalized fragments per kilobase of transcript per million mapped reads (FPKM) value of 0.1 or greater. Cuffdiff uses the statistical equation *T* = *E*[*log*(*y*)]∕*Var*[*log*(*y*)] where *y* is the ratio of the normalized counts between two conditions. Hence, a *t*-test was used to generate a *p*-value for differential expression. No threshold was set for the magnitude of differential expression given the lack of a priori knowledge regarding the biological impact of these RNA transcripts. Therefore, differential expression was defined using statistical criteria. Genes with a false discovery rate (*q*-value) less than 0.05 were considered significant. However, genes identified using the more lenient statistical threshold of *p* < 0.05 are also reported in the Supplementary Files.

### Weighted gene co-expression network analysis (WGCNA)

Weighted gene co-expression network analysis (WGCNA) is a systems biological technique used to identify networks of co-expressed genes as they relate to a phenotypic trait (Langfelder and Horvath, 2008). This approach calculates correlation coefficients between the expression values of genes and determines a connectivity measure (topological overlap) by summing their connection strengths with other genes. These calculations permit the clustering of genes into distinct modules based on their topological overlap. In order to determine if there is a relationship between the formed modules and a phenotypic trait, the first principle component (eigengene) of the modules can then be correlated with the phenotype data. With the inclusion of noncoding RNAs in this analysis, it is possible to identify noncoding RNAs showing highly correlated expression with protein coding genes important in neural differentiation and infer their functional properties through these associations.

As a result of their common use in neuroscience and the robust transcriptional changes observed in the present study, the SK-N-SH cell line was chosen for the construction of a weighted gene co-expression network. A signed co-expression network was built for SK-N-SH cells using the WGCNA package (version 1.46) in R. Normalized gene expression values were collected for each sample using Cuffdiff. The genes used in the co-expression network were restricted to those showing a minimum of 0.1 FPKM to reduce the amount of noise in the network. This resulted in 27,984 expressed genes. The WGCNA method creates an adjacency matrix from the gene correlation values using a power function. The power was selected from a scale free topology fit and a power of 20 was chosen for the network. The “blockwiseModules” function was utilized for network construction. To facilitate the formation of large and distinct modules, the minimum module size was set to 100 with the minimum module membership connectivity (kME) set at 0.7. Modules showing a correlation value of 0.75 were merged.

The eigengene of the formed modules was then extracted and a Pearson's correlation was calculated with the cell collection time. These calculations were used to identify biologically interesting modules for further analysis. Modules were selected for additional analysis if the correlations with cell collection time had a *p* < 0.1.

### Functional enrichment

In order to functionally annotate differentially expressed genes and gene co-expression modules of interest, the Database for Annotation, Visualization and Integrated Discovery (DAVID) (version 6.7; Dennis et al., 2003) was used to identify enriched gene ontology terms. Analyses were carried out using default options and redundant terms or terms with a Benjamini-Hochberg corrected *p* > 0.05 were removed.

### Disease-associated gene enrichment

To investigate whether differentially expressed genes are enriched in disease related gene sets, lists of genes implicated in autism spectrum disorder, Alzheimer's disease, and cancer were collected. Autism candidate genes were compiled from the Simons Foundation Autism Research Initiative (SFARI) AutDB database (Abrahams et al., 2013). This gene list was restricted to only include genes with SFARI gene rankings 1–4 (strong evidence—minimal evidence) and S (syndromic) as performed previously (Wilkinson et al., 2015). The list of genes implicated in Alzheimer's disease was obtained from ALZGENE (Bertram et al., 2007) and cancer related genes were collected from the Catalogue of Somatic Mutations in Cancer (COSMIC) (Futreal et al., 2004). Because most of the genes listed in these lists are protein coding, the number of expressed protein coding genes in SK-N-SH and ReNcell CX cells served as the background for enrichment tests. Statistics were analyzed using the two-tailed Fisher's exact test using the (fisher.test) package in R.

## Results

### Validation of gene expression in transfected cells

To ensure that transfection with an empty vector using Nucleofector technology does not produce an overall effect on the transcriptional profile of the cells, qPCR was performed on cells exposed to the transfection protocol as well as transfection naïve cells. Comparison of these experimental procedures indicated a significant Pearson's correlation coefficient for the level of gene expression changes between the 24 and 72 h time-points in both SK-N-SH (*R* = 0.76, *p* = 0.048) and ReNcell CX cells (*R* = 0.79, *p* = 0.036) (Supplementary Figure 1; Supplementary Table 2). Thus, exposure to transfection did not have an overall effect on gene expression in these cell lines.

### Neuronal morphology of differentiating cells

Images of SK-N-SH and ReNcell CX cells were captured prior to cell harvesting in order to characterize the cell morphology observed at the 24 and 72 h collection time-points. SK-N-SH cells showed distinct stages of neuronal maturation with small outgrowth of neurites evident at 24 h and longer, and more mature neurites present at 72 h (Supplementary Figure 2). ReNcell CX cells revealed a more rapid replication time and achieved a more mature neuronal morphology characterized by longer neurites at the 24 h time-point when compared to SK-N-SH cells. However, it was evident that ReNcell CX cells collected at the 72 h time-point were more mature than the cells collected at the earlier time-point (Supplementary Figure 2) indicating that these cells are in distinct stages of neural differentiation at these two collection time-points.

### Distribution of RNA-sequencing reads

**Figure 1:**
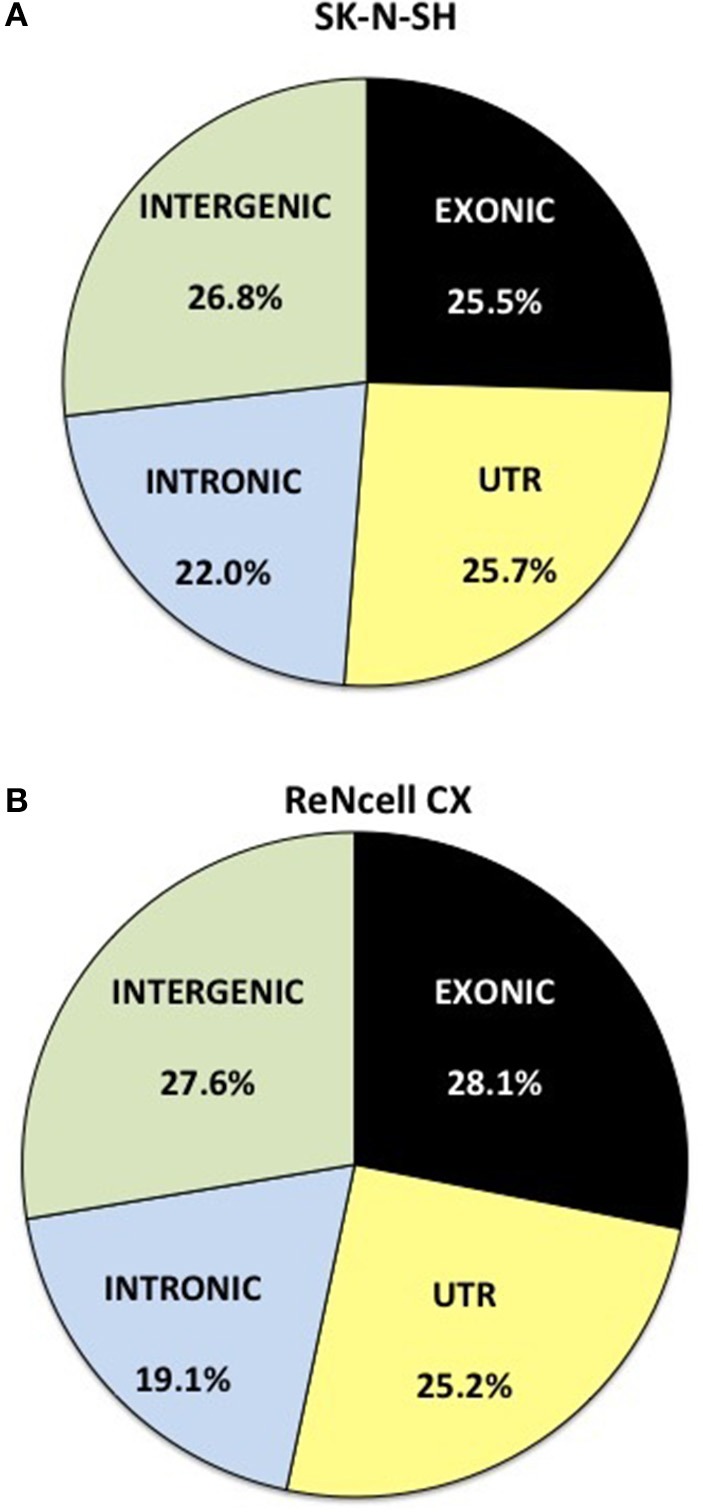
**Distribution of RNA sequencing reads in neural progenitor cell lines, SK-N-SH and ReNcell CX**. Reads were mapped to the transcriptome of ENSEMBL GrCH38 release 77. The reads were assigned as exonic (black), untranslated region (UTR) (light yellow), intronic (light blue), or intergenic (light green) regions. All replicates were averaged in **(A)** SK-N-SH and **(B)** ReNcell CX cell lines.

RNA-sequencing reads were aligned to the ENSEMBL GrCH38 version 77 genome and the distribution of reads was assessed. Reads obtained from SK-N-SH cells were mapped to mRNA with an average of 25.5% localizing to exonic regions and 25.7% mapping to untranslated regions (UTR) (Figure 1A). The remaining reads aligned to areas of the genome shown to harbor noncoding RNAs with 22.0 and 26.8% mapping to intronic and intergenic regions, respectively. ReNcell CX cells contained a similar expression profile with 28.1% of reads localizing to exonic regions (Figure 1B) and 25.2% mapping to the UTR. The remaining reads in ReNcell CX cells mapped to intronic (19.1%), and intergenic (27.6%) regions of the genome.

**Figure 2:**
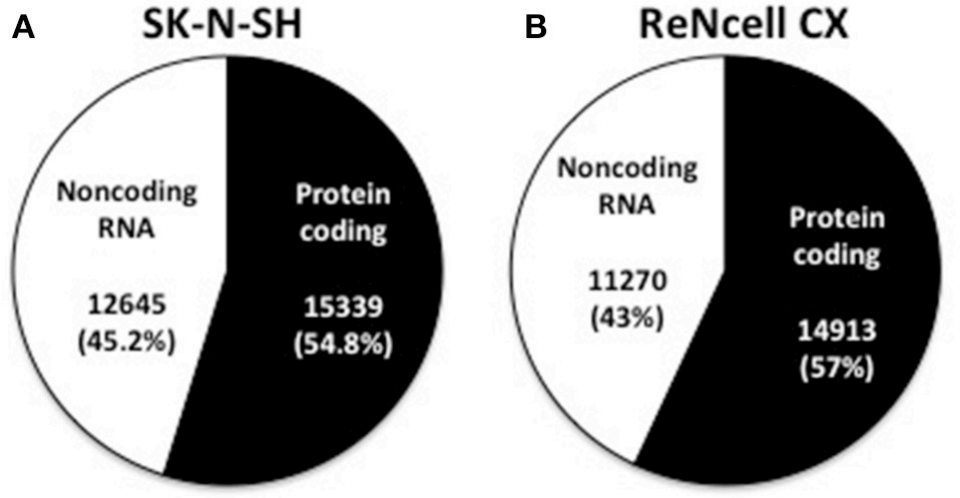
**Classification of expressed genes in human neural progenitor cell lines**. Expressed genes were classified as being either protein coding or noncoding RNA in both SK-N-SH **(A)** and ReNcell CX cells **(B)**.

The number of expressed genes (see Materials and Methods) in human neural progenitors cells was analyzed and resulted in SK-N-SH cells expressing 27,984 genes and 26,183 genes being expressed in ReNcell CX cells (Table 1). All expressed genes are listed in Supplementary Table 3. Genes were then classified based on their gene type and revealed that 54.8 and 57.0% of the genes in SK-N-SH and ReNcell CX cells, respectively, are protein coding (Figure 2). The remaining genes were found to be various classifications of noncoding RNAs (Table 1).

**Table 1:** **Characterization of expressed genes in human neural progenitor cells**.

**Cell line**	Number of expressed genes^*^	**Protein coding**	**Antisense**	**Pseudogene**	**lncRNA**	**pre-miRNA**	**Other noncoding RNA**
SK-N-SH	27984	15339 (54.8%)	3369 (12.0%)	3517 (12.6%)	2368 (8.5%)	375 (1.3%)	3016 (10.8%)
ReNcell CX	26183	14913 (57.0%)	3100 (11.8%)	2948 (11.2%)	2059 (7.9%)	390 (1.5%)	2775 (10.6%)

* *Minimum 0.1 fragments per kilobase of transcript per million mapped reads (FPKM)*.

*lncRNA, long noncoding RNA; pre-miRNA, pre-micro-RNA*.

The top 5% of genes showing the highest normalized expression values were identified (Supplementary Table 4). Noncoding RNAs comprised 6.6 and 6.7% of the genes showing the highest level of expression in SK-N-SH and ReNcell CX cells, respectively. Interestingly, a known long noncoding RNA shown to be important in regulating gene expression and synaptogenesis, *MALAT1*, is highly expressed in both cell lines along with other noncoding RNAs of unknown function (Bernard et al., 2010).

**Figure 3:**
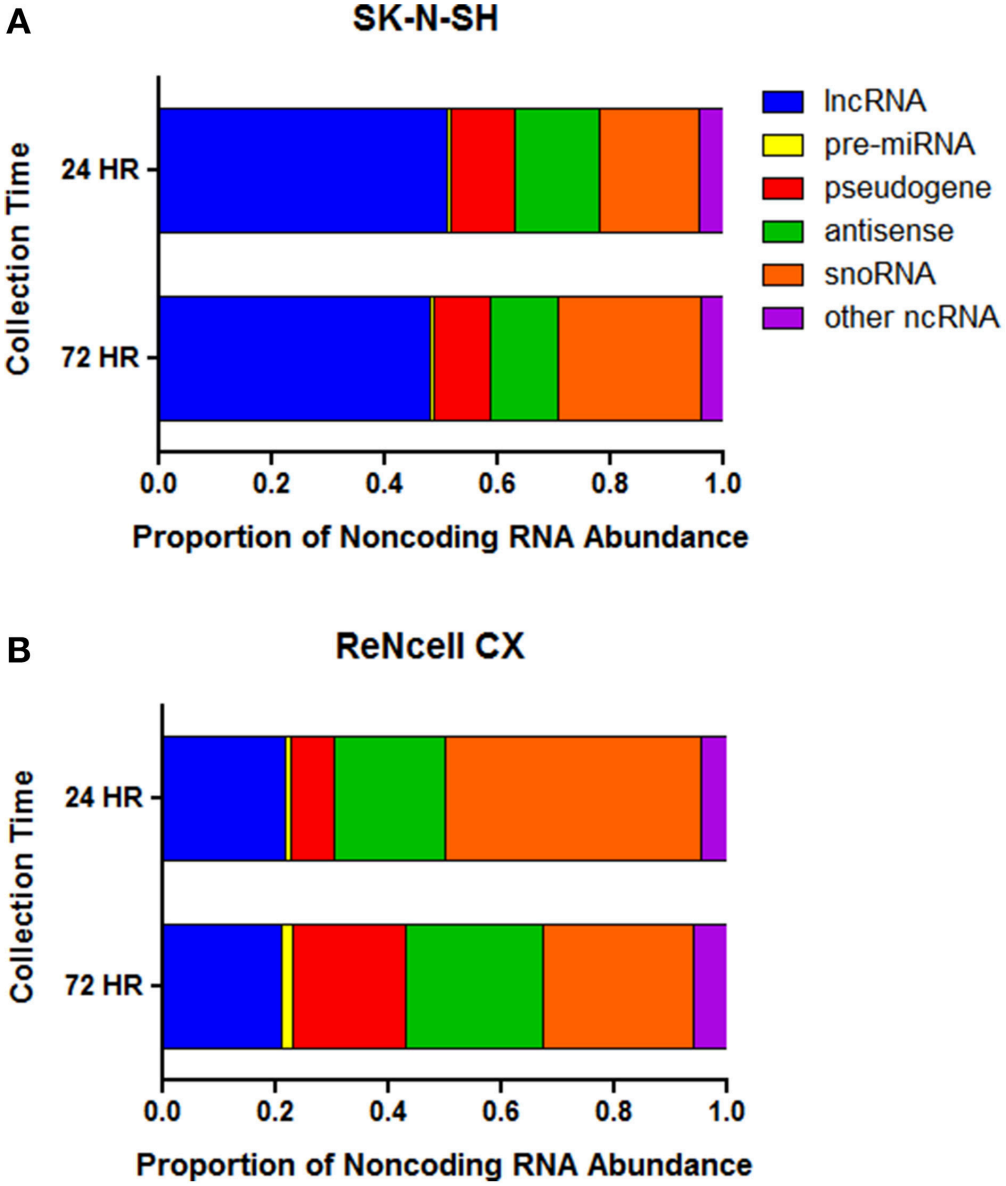
**Proportion of normalized expression values attributed to noncoding RNA subtypes**. The proportion of the FPKM values attributed to distinct classes of noncoding RNA in **(A)** SK-N-SH and **(B)** ReNcell CX cells. Long noncoding RNA (lncRNA), precursor microRNA (pre-miRNA), antisense transcript (antisense), small nucleolar RNA (snoRNA), other noncoding RNA (ncRNA).

The abundance of noncoding RNA subtypes was assessed in both cell lines. Long noncoding RNAs (lncRNA) account for the greatest proportion of the noncoding RNAs in SK-N-SH cells at both the 24 h (51.1%) and 72 h (47.9%) collection time-points (Figure 3A). The remaining expression of noncoding RNAs in SK-N-SH cells collected at 24 h was attributed to snoRNAs (17.7%), antisense transcripts (15.1%), pseudogenes (11.2%), precursor miRNAs (pre-miRNA) (0.8%), and other noncoding RNAs (4.1%). Cells collected at the more differentiated state (72 h) show slight shifts in the abundance patterns of snoRNAs (25.2%), antisense transcripts (12.1%), pseudogenes (9.9%), pre-miRNAs (0.7%), and other noncoding RNAs (4.2%).

The subtype of noncoding RNA showing the greatest abundance in ReNcell CX cells was snoRNAs representing 45.2 and 26.6% of the normalized expression values of noncoding RNAs in cells collected at 24 and 72 h, respectively (Figure 3B). The abundance of lncRNAs, antisense transcripts, pseudogenes, pre-miRNAs, and other noncoding RNAs in cells collected at the 24 h time-point represented 21.6, 19.8, 7.6, 1.1, and 4.7% of the expression, respectively. ReNcell CX cells collected at the more differentiated state show 21.2, 24.3, 20.1, 1.8, and 7.2% of the expression values of noncoding RNAs being attributed to lncRNAs, antisense transcripts, pseudogenes, pre-miRNAs, and other noncoding RNAs, respectively.

### Differential gene expression in neural differentiation

Both human neural progenitor cell lines were collected at two time-points of neural differentiation. Differential gene expression analysis revealed transcriptional changes between these time-points in both cell lines (Supplementary Table 5). All of the differentially expressed genes in both SK-N-SH and ReNcell CX cells are listed in Supplementary Table 6 with their associated *q*-values and more statistically lenient *p*-values.

**Figure 4:**
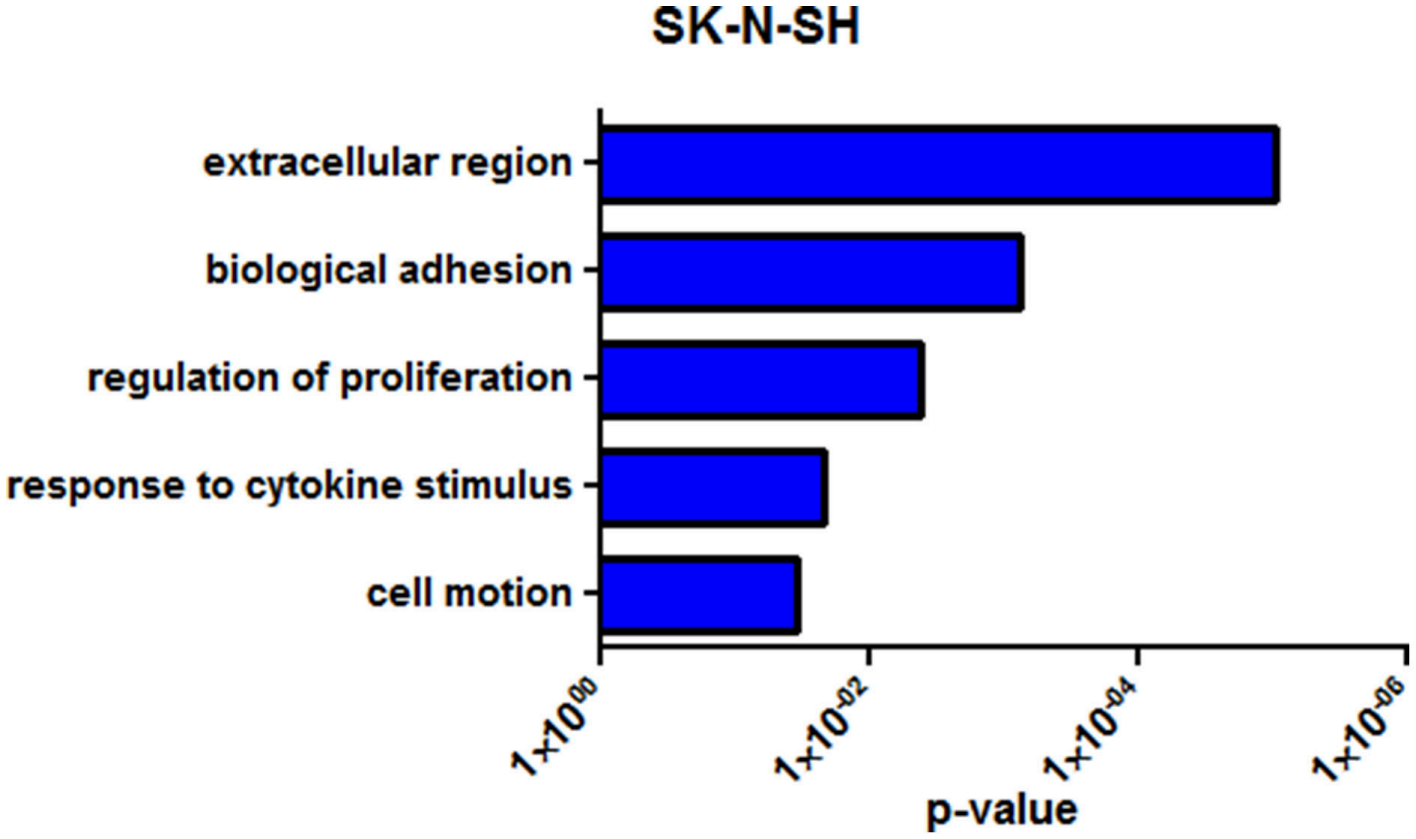
**Representative enrichment of gene ontology among differentially expressed genes (***q*** < 0.05)**. GO enrichment analysis of SK-N-SH. *P*-value represents Benjamini-Hochberg correction for multiple tests.

SK-N-SH cells revealed 222 differentially expressed genes (*q* < 0.05) between the two collection times with 105 (47.3%) being upregulated in the more differentiated state (72 h). Noncoding RNAs represented 4.5% of the differentially expressed genes in SK-N-SH cells. Functional enrichment in gene ontology terms was analyzed in differentially expressed protein coding genes in SK-N-SH cells and revealed enrichment in terms such as biological adhesion, regulation of cell proliferation, and cell motion (Figure 4) (Supplementary Table 7). An exploratory analysis was run using a statistical threshold of *p* < 0.05, and identified 1284 differentially expressed genes with 11.4% being noncoding RNA. Genes showing differential expression using the more lenient statistical threshold demonstrate functional enrichment in the same gene ontology terms revealed using the false discovery rate calculation (Supplementary Figure 3) (Supplementary Table 7).

ReNcell CX cells revealed differential expression in only 19 genes with 14 (73.7%) of these genes being upregulated at the 72 h time-point. All of the differentially expressed genes were identified as protein coding with functional enrichment in the gene ontology term relevant to a response to oxidative stress (Supplementary Table 7). To broaden this search, the *p*-value calculation was used and revealed differential expression in 534 genes with 20 (3.7%) being identified as noncoding RNA. Overall, these genes show functional enrichment in GO terms relevant to processes such as chromatin assembly, protein-DNA complexes, and biological adhesion (Supplementary Figure 3; Supplementary Table 7).

Investigation of the differentially expressed genes revealed 7 genes showing altered expression in both cell lines as they differentiate into cortical projection neurons. All of these genes were identified as protein coding genes. In an exploratory analysis, 106 genes were identified as being differentially expressed in both cell lines when using the more lenient statistical cutoff for differential expression with 4 of these genes being identified as noncoding RNA.

### Enrichment of disease-associated genes

Differentially expressed genes from the two cell lines were analyzed for enrichment in lists of genes implicated in a neurodevelopmental disorder (autism spectrum disorder), a neurodegenerative disorder (Alzheimer's disease), and cancer. Genes differentially expressed in SK-N-SH cells were shown to be trending for enrichment in the gene list for autism spectrum disorder (ASD) (*p* = 0.061, *OR* = 1.959) while showing no enrichment in genes implicated in either Alzheimer's disease (*p* = 0.118, *OR* = 1.460) or cancer (*p* = 0.072, *OR* = 1.623). However, a significant enrichment was observed in the gene list for autism spectrum disorder (*p* = 0.0454, *OR* = 1.437) when using the less stringent statistical threshold. No statistically significant enrichment was observed in Alzheimer's disease (*p* = 0.575, *OR* = 0.982) or cancer (*p* = 0.279, *OR* = 1.101). The autism-associated genes showing differential expression in SK-N-SH cells are listed in Table 2.

**Table 2:** **ASD-associated genes differentially expressed in SK-N-SH cells**.

**ASD-associated gene**	**Fold change**	Locus^*^	***p*-value**	***q*-value**
*CNTNAP4*	8.62	16:76277277-76928169	5.00E-05	6.07E-3
*CNTNAP5*	3.73	2:124025286-124915287	5.00E-05	6.07E-3
*CNTNAP2*	3.62	7:146116360-148420998	5.00E-05	6.07E-3
*ICA1*	2.93	7:8113183-8344516	5.00E-05	6.07E-3
*FHIT*	2.38	3:59749309-61251459	4.77E-02	5.91E-1
*CHRNA7*	2.37	15:32030487-32172521	1.50E-04	1.52E-2
*DPP6*	2.33	7:153887096-154894285	2.00E-04	1.86E-2
*CACNA1C*	2.13	12:1946053-2697950	3.00E-03	1.25E-1
*CNTN4*	1.99	3:2098812-3061145	3.15E-03	1.28E-1
*PARK2*	1.90	6:161347419-163315492	2.17E-02	4.07E-1
*SEMA5A*	1.90	5:9001773-9546075	2.75E-03	1.17E-1
*RIMS3*	1.87	1:40620678-40665657	9.00E-04	5.76E-2
*CADM1*	1.85	11:115169217-115504957	1.25E-03	7.22E-2
*MYO16*	1.81	13:108596151-109208007	6.25E-03	1.99E-1
*RPS6KA2*	1.79	6:166409363-166957191	2.18E-02	4.07E-1
*MYT1L*	1.75	2:1789112-2331260	8.25E-03	2.36E-1
*ASXL3*	1.74	18:33578614-33751192	7.75E-03	2.28E-1
*AUTS2*	1.63	7:69598918-70793068	6.55E-03	2.04E-1
*NRXN2*	1.61	11:64606173-64723188	4.14E-02	5.53E-1
*KDM5B*	1.55	1:202724490-202809470	1.22E-02	2.98E-1
*GABRB3*	1.55	15:26543545-26949207	1.49E-02	3.35E-1
*AGAP1*	1.53	2:235494088-236131800	4.32E-02	5.65E-1
*DPYD*	−1.43	1:97077742-97921049	4.23E-02	5.59E-1
*RAPGEF4*	−1.60	2:172677140-173052893	6.35E-03	2.00E-1
*MET*	−1.65	7:116672389-116798386	8.45E-03	2.39E-1
*TCF7L2*	−1.68	10:112950249-113167678	4.16E-02	5.53E-1
*NRP2*	−1.73	2:205681989-205798133	8.00E-03	2.31E-1
*ANXA1*	−1.81	9:73151756-73170393	2.45E-03	1.08E-1
*PCDH9*	−1.83	13:66302833-67230445	1.92E-02	3.84E-1
*IL1RAPL1*	−2.55	X:28587398-29956723	4.01E-02	5.46E-1
*ITGB3*	−2.73	17:47253845-47441312	2.00E-04	1.86E-2
*NLGN1*	−3.59	3:173396283-174286644	5.00E-05	6.07E-3
*TBX1*	−7.32	22:19756702-19854939	3.95E-02	5.42E-1

*ASD, autism spectrum disorder*.

* *Refers to Ensembl GrCH38.77*.

Enrichment analysis was also performed on genes showing altered expression in ReNcell CX cells. Contrary to the results found in SK-N-SH cells, differentially expressed genes in the ReNcell CX cell line failed to reach statistical significance for enrichment in gene lists for ASD (*p* = 1.00, *OR* = 1.00), Alzheimer's disease (*p* = 0.214, *OR* = 2.466), and cancer (*p* = 1.00, *OR* = 1.00). Enrichment remained nonsignificant for autism spectrum disorder (0.901, *OR* = 0.659), Alzheimer's disease (*p* = 0.560, *OR* = 0.984), and cancer (*p* = 0.574, *OR* = 0.977) when analyzing the genes showing differential expression using the more lenient statistical cutoff.

### Weighted gene co-expression network analysis

Identifying noncoding RNAs regulating complex biological processes remains a difficult challenge due to the vast landscape of uncharacterized noncoding RNAs. To narrow the list of noncoding RNAs possibly regulating neural differentiation, a weighted gene co-expression network was constructed to form distinct modules of protein coding and noncoding genes showing highly correlated expression patterns. This network construction allows for the identification of transcriptional networks possibly being regulated by these noncoding RNAs.

**Figure 5:**
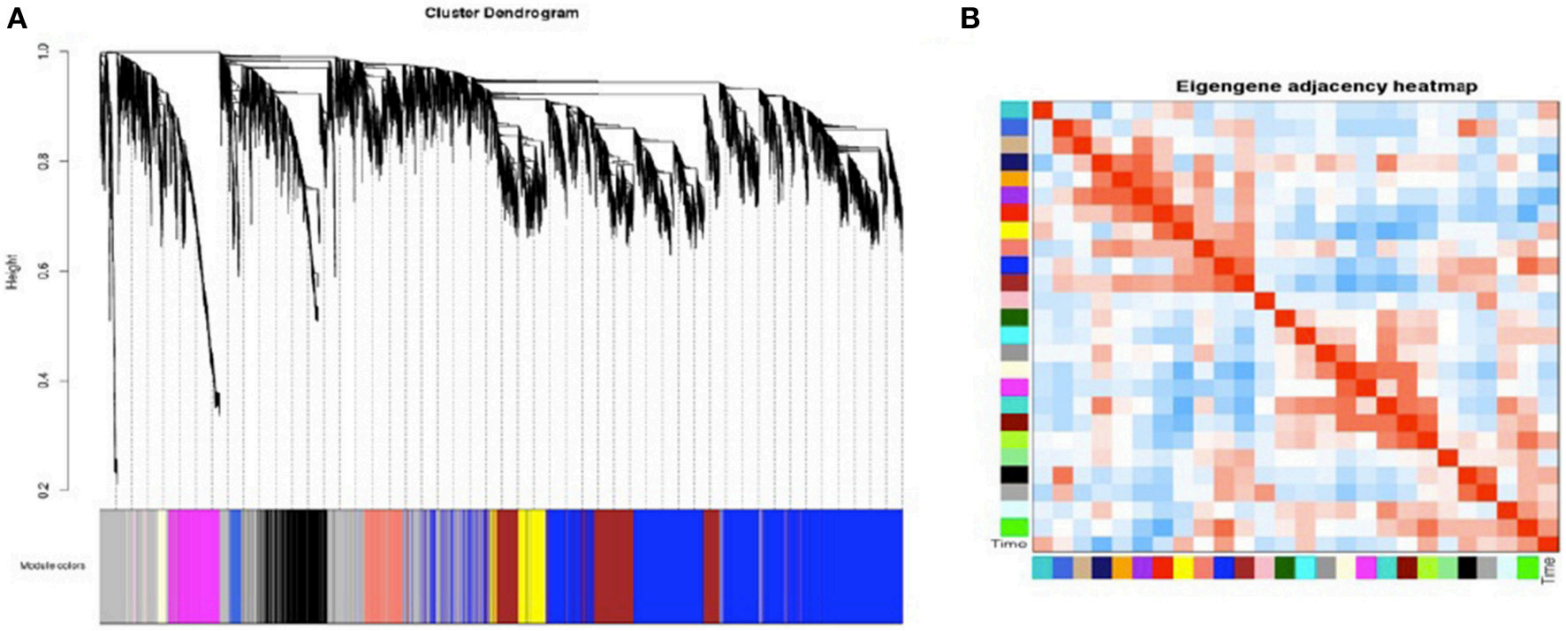
**Weighted Gene Co-Expression Network Analysis of SK-N-SH neural progenitor cells. (A)** Gene dendrogram of co-expressed genes in SK-N-SH cells. Weighted Gene Co-Expression Network Analysis (WGCNA) revealed 25 modules of co-expressed genes not including the gray module. **(B)** Heatmap indicating module eigengene similarity and relation to cell collection time.

SK-N-SH cells were selected for construction of a gene co-expression network due to the robust transcriptional changes observed as a result of neural differentiation. A signed network was constructed using the 27,984 expressed genes containing a minimum FPKM value of 0.1. The constructed network revealed 25 distinct modules of co-expressed genes (Figure 5A) and these modules were each assigned a color (Supplementary File 3). Four modules were identified as being associated with cell collection time (Figure 5B) and were selected for further analysis. The modules meeting criterion were the midnight blue, purple, green, and blue modules (*p* = 0.02, *p* = 0.03, *p* = 0.05, *p* = 0.09, respectively).

### Characteristics of neural differentiation associated modules

**Figure 6:**
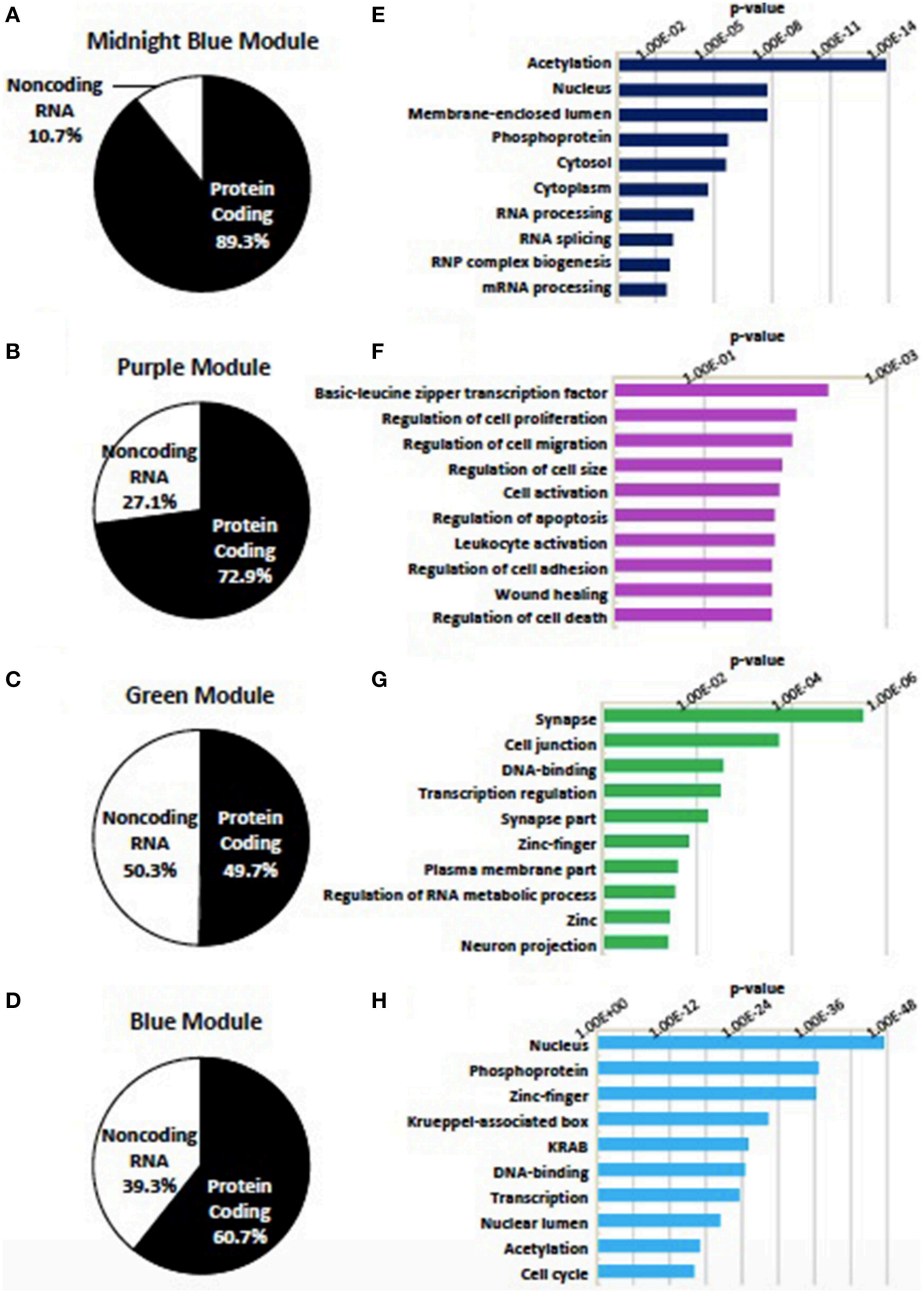
**Modules identified as being associated with differentiation status**. Four modules (midnight blue, purple, green, blue) were found to be associated with the status of neural differentiation in SK-N-SH cells. **(A–D)** Distribution of genes within the **(A)** midnight blue, **(B)** purple, **(C)** green, and **(D)** blue modules as being either protein coding or noncoding RNA. Noncoding RNA is present in all four modules ranging from 10.7 to 49.7% of genes in associated modules. **(E–H)** Representative enrichment of gene ontology among all genes in the **(E)** midnight blue, **(F)** purple, **(G)** green, and **(H)** blue modules. *p*-value represents Benjamini-Hochberg correction for multiple tests.

Genes assigned to the midnight blue, purple, green, and blue modules were first divided as being either protein coding or noncoding RNA. Noncoding RNA represented 10.7, 27.1, 49.7, and 39.3% of the genes assigned to these modules, respectively (Figures 6A–D). In agreement with their association with neural differentiation, the four modules show an enrichment of genes within GO categories relevant to the synapse, transcription, chromatin remodeling, and the regulation of cell proliferation and cell size (Figures 6E–H) (Supplementary Table 8).

## Discussion

The differentiation of pluripotent stem cells into human cortical projection neurons involves a complex network of coordinated changes in gene expression. With the growing appreciation that noncoding RNAs play critical roles in the regulation of gene expression, it is likely that noncoding RNAs are crucial for proper neurogenesis and differentiation. The present study used RNA sequencing to detect noncoding RNAs that are expressed in the human neural progenitor cells, SK-N-SH and ReNcell CX, as they differentiate into cortical projection neurons. The transcriptional profiles of these cells contain a large proportion of noncoding RNAs. It has been postulated that the rise in non-protein coding regions of the genome corresponds to the evolutionary development of more complex organisms (Taft et al., 2007; Liu et al., 2013). As such, the human transcriptome, particularly in the brain, has been shown to express a large amount of these regulatory transcripts (Barry, 2014). In fact, previous research has suggested that 64% of the transcriptional reads in the human brain are noncoding RNA with smaller proportions being evident in other tissues (Kapranov et al., 2010). Consistent with these previous findings, approximately half of the expressed genes in the two human neural progenitor cells used in this study were found to be protein coding and only 25–28% of raw sequencing reads were shown to align to exonic regions of the genome. Interestingly, a proportion (6.6–6.7%) of the genes showing the highest expression in these cell lines are noncoding RNA. One of the noncoding RNAs showing high expression in both cell lines is *MALAT1*. Previous research has found this long noncoding RNA to be highly expressed in the brain and to hold key functional roles in gene regulation and synaptogenesis (Bernard et al., 2010). Long noncoding RNAs (including lncRNA, antisense transcripts, and pseudogenes) were shown to account for a large proportion of the normalized reads of noncoding RNAs in both neural progenitor cell lines. Interestingly, these long noncoding RNAs show greater abundance than pre-miRNAs, which are widely recognized for their regulatory roles on gene expression (Vidigal and Ventura, 2015), suggesting their importance in the regulation of gene networks in neural cells.

Both cell lines investigated in this study showed differential expression of both protein coding and noncoding RNAs as the cells differentiate. Interestingly, the differentially expressed genes were significantly enriched in gene ontology terms important in neural differentiation. SK-N-SH cells portrayed a more robust transcriptional change between the 24 and 72 h collection time-points compared to ReNcell CX cells. As a result, several of the gene ontology terms identified using the differentially expressed genes identified using the less stringent statistical threshold (*p*-value) were also observed using the false discovery rate calculation (*q*-value) in SK-N-SH cells but not in ReNcell CX cells (Supplementary Table 7). The less dramatic transcriptional shift in ReNcell CX cells is likely a result of the observed differences in the replication time and cell morphology observed in these cells. ReNcell CX cells replicated at a more rapid rate and achieved a more mature neural phenotype at the 24 h time-point compared to SK-N-SH cells. It is likely that a more pronounced transcriptional change would be observed in ReNcell CX cells at an earlier time-point before a mature neuronal morphology is achieved. Only four noncoding RNAs were found to be differentially expressed in both cell lines when using the less stringent statistical cutoff. These results suggest that the two cell lines investigated were likely in differing stages of differentiation and that noncoding RNAs likely show cell type specificity, an idea that has been proposed in previous studies (Cheng et al., 2005; Tsoi et al., 2015). However, further experimentation is needed to address these questions.

SK-N-SH cells revealed dramatic changes in the transcriptional profile of cells collected at the 24 and 72 h time-points. One of the noncoding RNAs showing altered expression in this cell line was *H19*. This long noncoding RNA has been extensively studied in cancer and is suggested to act as a precursor to several miRNAs and have downstream effects on cell proliferation and insulin-like growth factor signaling (Cai and Cullen, 2007; Keniry et al., 2012). While this known noncoding RNA may provide insight into the biological processes being modulated in these differentiating cells, the majority of the differentially expressed noncoding RNAs have unknown functions and will require further experimentation to identify their functional properties.

Interestingly, differentially expressed protein coding genes in SK-N-SH cells were found to be enriched in genes implicated in autism spectrum disorder, while showing no enrichment in genes linked to a neurodegenerative disorder or cancer implicating these genes in early neurodevelopmental processes. In order to identify the noncoding RNAs possibly regulating the expression of these gene networks in SK-N-SH cells, a gene co-expression network was created. This systems biological approach creates modules of genes showing highly correlated expression values. By incorporating noncoding RNAs in the analysis, this method allows for a narrowing of the vast landscape of these regulatory RNAs and provides the opportunity to imply function through their co-expressed protein coding genes. This analysis identified four modules associated with neural differentiation status and gene ontology of the protein coding genes in these modules were enriched in terms shown to be important in this biological process (Gurok et al., 2004; Fathi et al., 2011). Additionally, each of these modules was shown to contain large proportions of noncoding RNAs. For example, the long noncoding RNA, *TUNAR*, was identified in the blue module along with genes associated with DNA-binding and transcriptional processes. *TUNAR* (*TCL1* Upstream Neural Differentiation-Associated RNA) has previously been found to regulate pluripotency and is required for neural differentiation through its recruitment of RNA binding proteins to *NANOG, SOX2*, and *FGF4* promoters (Lin et al., 2014). Additionally, *MIR137HG*, was located in the green module which is characterized by genes associated with the synapse and transcriptional regulation. *MIR137HG* is a precursor to the miRNA, *MIR137*, known to have regulatory properties important for proper neuronal and dendritic development (Smrt et al., 2010; Szulwach et al., 2010; Tarantino et al., 2010; Sun et al., 2011). This miRNA has also been implicated in several neuropsychiatric conditions such as autism spectrum disorder and schizophrenia (Collins et al., 2014; Devanna and Vernes, 2014). Therefore, it is likely that the noncoding RNAs identified in the neural differentiation-associated modules hold regulatory properties important in this biological process and alterations in their expression may underlie the pathogenesis of neurodevelopmental disorders. However, further experiments are needed to investigate the individual functions of these RNA transcripts.

The present study demonstrates the vast landscape of noncoding RNAs that are expressed in human cortical neurons. Additionally, dramatic shifts in gene expression occur as these cells undergo early differentiation and indicate changes in genes linked in autism spectrum disorder. The identification of noncoding RNAs showing highly correlated expression patterns with protein coding genes using WGCNA lays the foundation for future explorations into the functional properties of these non-protein coding transcripts.

### Conflict of interest statement

The authors declare that the research was conducted in the absence of any commercial or financial relationships that could be construed as a potential conflict of interest.
