# Supplementary material for: Noncoding RNA in the transcriptional landscape of human neural progenitor cell differentiation
Source: Front Neurosci. 2015 Oct 23;9:392. doi: 10.3389/fnins.2015.00392 (PMC4615820; doi:10.3389/fnins.2015.00392)
Supplement: Supplementary file 9 [file Image1.PDF]

Supplementary Figure 1. Transfection with empty vector has no overall impact of transcriptional profile. qPCR validation of the similar gene expression patterns observed in select protein coding and noncoding RNAs in **(A)** SK-N-SH cells and **(B)** ReNcell CX cells.

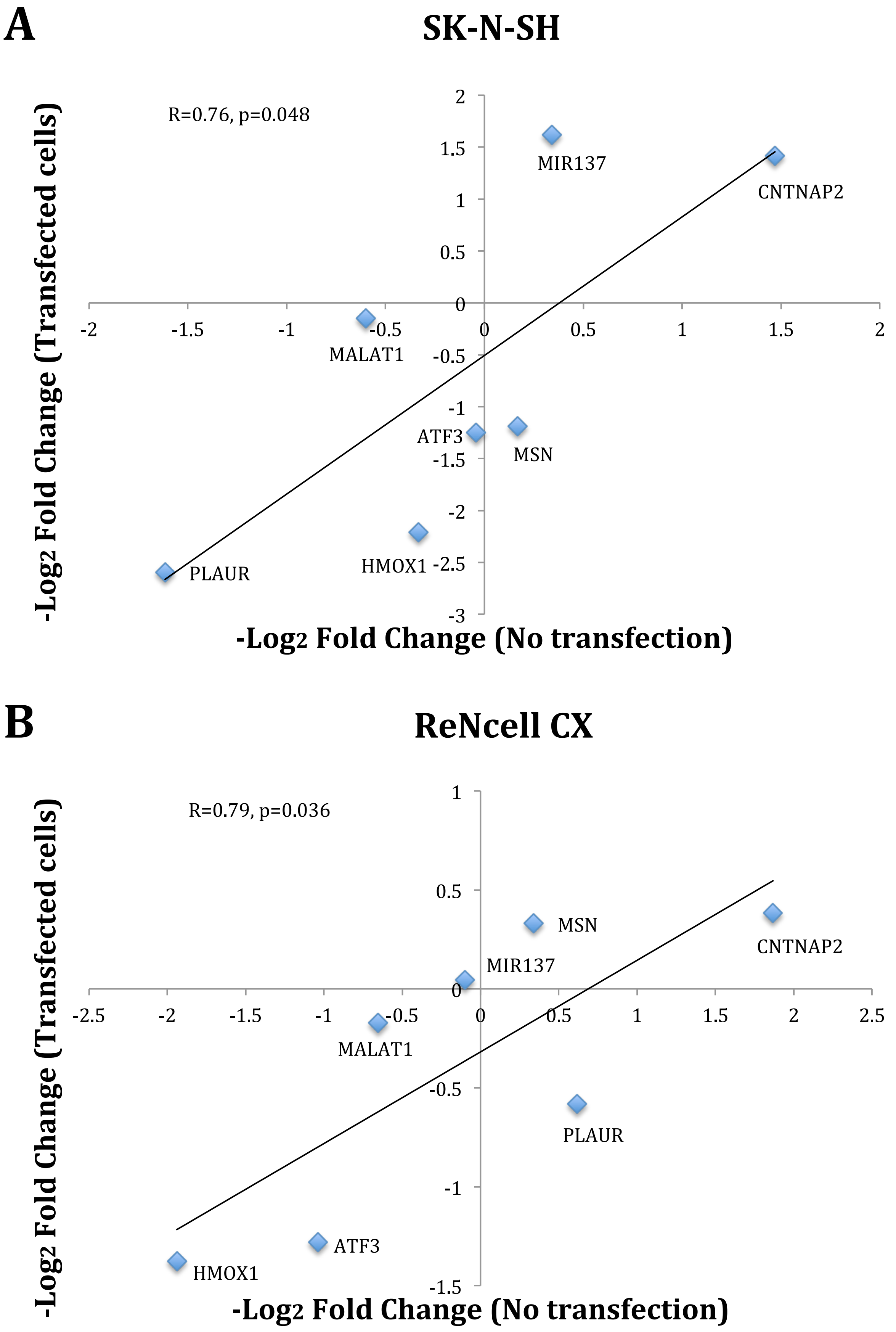
A
SK-N-SH
R=0.76, p=0.048
MIR137
CNTNAP2
MALAT1
ATF3
MSN
HMOX1
PLAUR
-Log2 Fold Change (Transfected cells)
-Log2 Fold Change (No transfection)
B
ReNcell CX
R=0.79, p=0.036
MSN
CNTNAP2
MIR137
MALAT1
PLAUR
ATF3
HMOX1
-Log2 Fold Change (Transfected cells)
-Log2 Fold Change (No transfection)
