# Supplementary material for: Noncoding RNA in the transcriptional landscape of human neural progenitor cell differentiation
Source: Front Neurosci. 2015 Oct 23;9:392. doi: 10.3389/fnins.2015.00392 (PMC4615820; doi:10.3389/fnins.2015.00392)

Supplementary Figure 2. Cell morphology of human neural progenitor cells undergoing differentiation. Representative brightfield images of SK-N-SH and ReNcell CX cells at the 24 and 72 h collection time-points. Scale bar represents 50 microns.

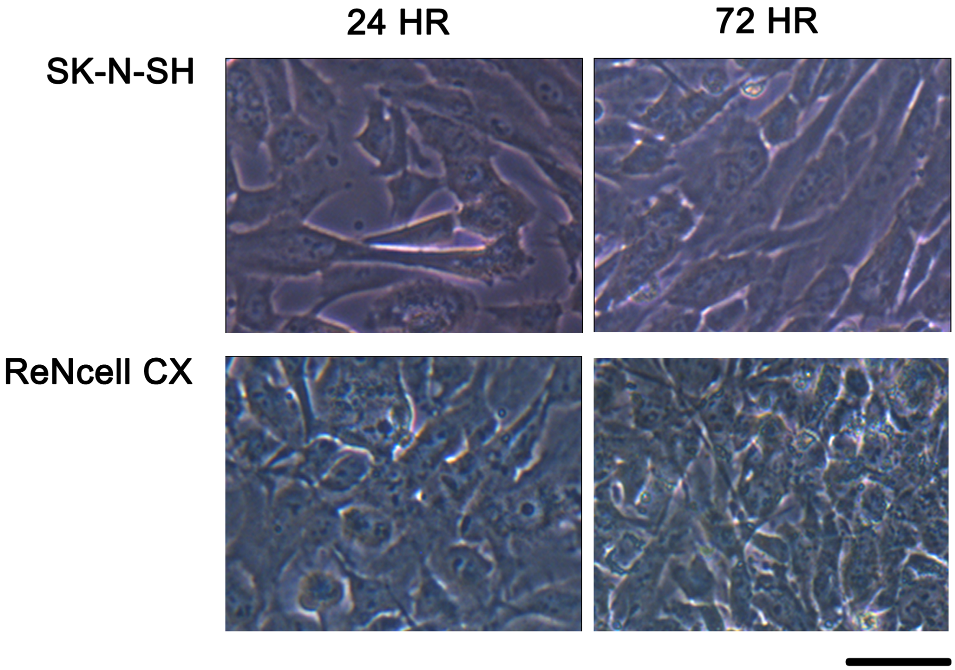

Supplement: Supplementary file 10 [file Image2.PDF]
