# Supplementary material for: Noncoding RNA in the transcriptional landscape of human neural progenitor cell differentiation
Source: Front Neurosci. 2015 Oct 23;9:392. doi: 10.3389/fnins.2015.00392 (PMC4615820; doi:10.3389/fnins.2015.00392)

Supplementary Figure 3: Representative enrichment of gene ontology among differentially expressed genes. GO enrichment analysis of SK-N-SH **(A)** and ReNcell CX cells **(B)**.

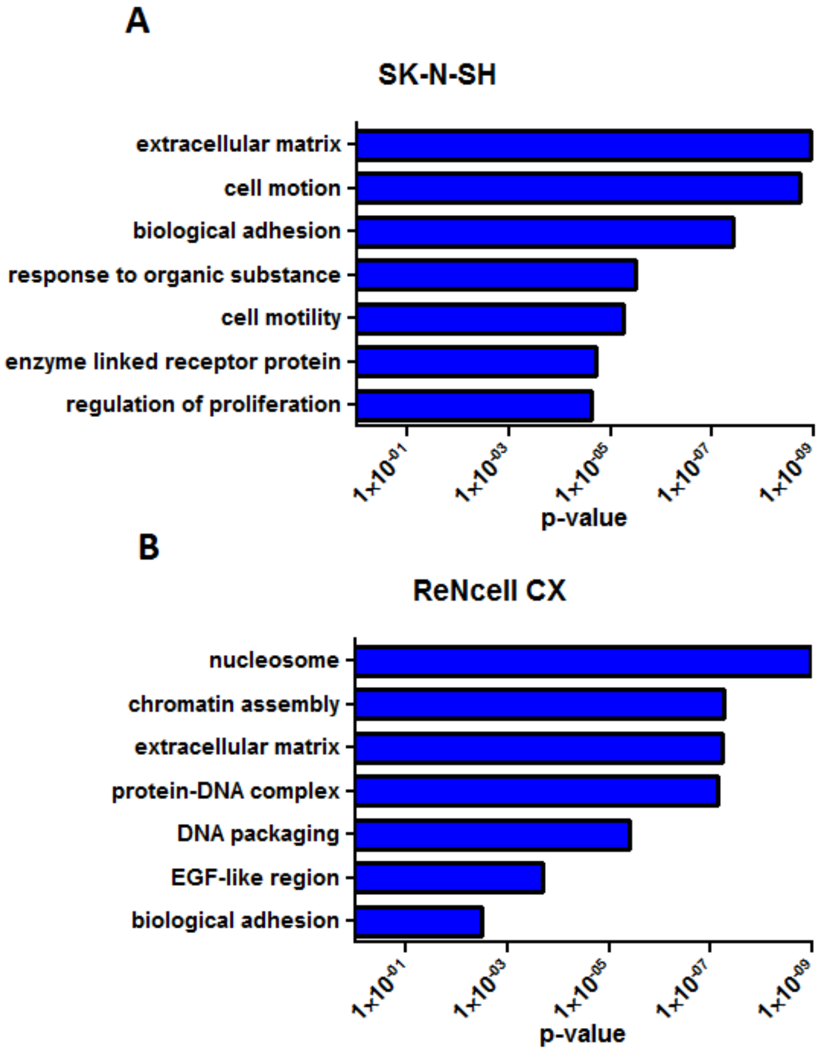

Supplement: Supplementary file 11 [file Image3.PDF]
